# Beyond the Binary: Advancing Gender-Inclusive Data Policy for Health Equity in the US National Provider Identifier System

**DOI:** 10.1089/heq.2025.0059

**Published:** 2025-06-12

**Authors:** Jeremy W. Jacobs, Shazia S. Khan, Laura D. Stephens, Garrett S. Booth

**Affiliations:** ^1^ Department of Pathology, Microbiology, & Immunology, Vanderbilt University, Nashville, Tennessee, USA.; ^2^ Departments of Pathology and Laboratory Medicine, Yale School of Medicine, New Haven, Connecticut, USA.; ^3^ Department of Pathology, University of California San Diego, La Jolla, California, USA.

**Keywords:** gender, sex, equity, diversity, inclusion, databases, health care, health policy

## Abstract

Achieving health equity requires data systems that recognize and reflect provider diversity. The National Provider Identifier (NPI) system underpins United States health care administration, yet its gender data standards remain outdated, conflating sex and gender and lacking inclusive options. These deficiencies undermine research, equity initiatives, and the visibility of transgender and nonbinary providers. In an era of growing political hostility to diversity, administrative neutrality is insufficient. The National Plan and Provider Enumeration System must establish itself as a model of gender-inclusive policy by separating sex and gender variables, expanding identity categories, and implementing transparent, regularly updated standards grounded in science.

## Introduction

Equity in health care must begin with equity in data. The National Plan and Provider Enumeration System (NPPES), which assigns a National Provider Identifier (NPI) to every United States (US) health care provider, might seem like an arcane administrative feature of the US health care system, but it is foundational to the infrastructure of health care delivery, billing, and workforce research. Established by the US Centers for Medicare and Medicaid Services (CMS) under the Administrative Simplification provisions of the Health Insurance Portability and Accountability Act (HIPAA), the NPI is a unique 10-digit identifier assigned to each health care provider.^1^ Its purpose: to streamline the exchange of electronic health information across payers, providers, and systems. Yet, embedded in this foundational system is a flawed demographic variable—“gender”—whose vague definition and limited options belie the complexity and diversity of the health care workforce.

Until April 2024, the NPPES permitted only two gender selections for an individual’s NPI: male and female.^2^ Following advocacy and policy pressure, CMS expanded these to include “unspecified or another gender identity” (X) and “undisclosed” (U).^2^ While a welcome step, this update falls short. The system still conflates sex and gender, lacks clarity on what each variable represents, and fails to capture the range of gender identities recognized in modern clinical and policy contexts. The implications are far from symbolic. Inaccurate gender data can undermine workforce analytics, weaken data-driven equity initiatives, and reinforce systemic barriers for transgender and nonbinary clinicians, leading to downstream inequities in workforce representation and patient care access.

As legislative and cultural attacks on gender diversity intensify in the US, the health care workforce and its associated data systems must assert their role as stewards of accuracy, inclusion, and professional legitimacy. This Commentary examines the shortcomings of the current NPI gender framework, articulates the risks it poses, and proposes reforms that would align it with both evidence and equity.

## Sex vs. Gender: Why the Distinction Matters

The conflation or interchangeable use of sex and gender is more than a semantic oversight—it is a policy failure with measurable consequences. Biological sex refers to physical and genetic traits typically categorized as male or female.^3–5^ Gender, by contrast, is a social construct encompassing norms, behaviors, roles, and attributes that a given society associates with individuals.^3–5^ Gender identity is self-identified, may evolve over time, and does not necessarily correspond to societal expectations based on biological sex.^3–5^ Leading medical and policy institutions—including the US National Academies, American Medical Association, and the Council of Europe—recognize this distinction and emphasize the need for administrative systems to reflect it.^3–5^

Yet the NPI system lags behind. Though the CMS documentation labels its demographic field as “gender,” the available selections (male, female, X, and U) do not map clearly onto either gender identity or biological sex. For example, a transgender woman assigned male at birth has no clear way to report both her biological sex and gender identity. This ambiguity leads to misclassification, as evidenced by early studies showing inconsistent uptake of the X and U options and confusion among health care providers.^2^ In their analysis of the NPPES, Corman and Przedworski remarked that “potential misclassification (of a clinician’s gender) may have occurred if clinicians misunderstood gender options,”^2^ underscoring the confusion and errors that can arise from ambiguous or misleading terminology.

This problem is not theoretical. Workforce research, such as studies examining pay equity, specialty representation, and workplace discrimination, increasingly relies on gender-disaggregated data. Inaccuracies or gaps in NPI data compromise these analyses and the policies they inform.^6–9^ Moreover, EHR systems, credentialing bodies, and public health researchers often draw from NPI data, meaning its shortcomings ripple across the entire health ecosystem.^10,11^

## The Real-World Impact on Gender-Diverse Providers

Administrative data systems shape not only what is measured but also who is recognized—and who is invisible. When systems like the NPI neglect to offer inclusive gender identity options, they send a message: that transgender and nonbinary providers do not count. This has direct implications for equity in workforce representation, institutional accountability, and provider well-being.

First, it exacerbates professional marginalization. Without accurate demographic data, institutions struggle to benchmark diversity, assess equity, or respond to discrimination. Second, it undermines mental health. Research shows that misgendering and the inability to self-identify in official records are associated with increased rates of anxiety, depression, and suicidality among gender-diverse individuals.^12^ Further, incongruence between identification documents and gender identity has been associated with worsened mental health outcomes, including elevated levels of anxiety and depression.^13^ Finally, in today’s politicized environment, the absence of inclusive federal standards leaves gender-diverse providers more vulnerable to hostile legislation. For example, Executive Order 14168, issued in January 2025,^14^ seeks to remove references to gender diversity in federal documentation under the banner of restoring “biological truth.” Without a countervailing federal standard grounded in science and equity, administrative silence becomes complicity. Thus, administrative neutrality is not sufficient; proactive inclusion is necessary.

The significance of this issue is evident given that, as of 2022, approximately 5.1% of US adults aged 18–30 identified their gender as different from their sex assigned at birth.^15^ While comprehensive data on transgender and nonbinary health care providers in the US are lacking, demographic trends suggest their representation in the workforce will continue to grow.^16^ Without precise and inclusive data collection, health care systems risk failing to recognize and support an increasingly diverse provider population, ultimately affecting workforce policies, equity initiatives, and patient care.

## Missed Opportunity: The 2024 Update

The April 2024 revision to the NPI gender field was an opportunity to lead. Instead, it defaulted to minimalism. Rather than modeling best practices—such as those recommended by the National Academies^5^ or aligning with systems like those used in the Veterans Health Administration^17^—which separately capture sex assigned at birth and current gender identity—CMS adopted a vague and internally inconsistent schema. The result: an appearance of progress without the functionality to match.

This form of performative inclusion, which offers symbolic change without structural reform, is common in DEI policy under political pressure. However, for datasets as integral as the NPI, with legal, financial, and reputational implications, such half-measures are not only insufficient; they are dangerous.

## Recommendations: Four Steps Toward Integrity and Inclusion

To transform the NPPES and NPI into a model of inclusive and accurate demographic data collection, we recommend CMS undertake the following reforms:

### Separate Sex and Gender as Distinct Fields

Demographic forms should include two clearly labeled fields: one for biological sex (with options such as male, female, and intersex) and one for gender identity (with expanded options). This separation enhances clarity, research utility, and individual autonomy.

### Expand Gender Identity Options

Options should include, at a minimum: woman, man, transgender woman, transgender man, nonbinary, genderqueer, agender, and self-describe (with free text). These categories should be based on consultation with medical, sociological, and community stakeholders to better reflect the full spectrum of gender diversity.

### Provide Definitions and Education

CMS should publish definitions for each demographic term and offer guidance for health care providers completing or updating their NPI registration. This minimizes misclassification and confusion and affirms the system’s legitimacy.

### Commit to Regular Review and Update

As gender identity language evolves, so too should administrative systems. Periodic reviews, which may be undertaken by a standing advisory group, could ensure the NPPES (and other federal data systems) remains aligned with current medical and social sciences and policy standards.

## Equity Impact

Improving gender representation in administrative data systems has wide-reaching implications. Accurate and inclusive NPI records would enable better research into workforce disparities, facilitate equitable policy development, and support gender-diverse providers in accessing professional opportunities. Moreover, studies indicate that transgender and gender-diverse patients often prefer providers who share or affirm their identities,^18^ making inclusive data a driver of better patient care.

## Policy Implications

In the current climate, where equity and diversity initiatives face increasing scrutiny, systems like the NPPES must lead by example. Failing to provide inclusive gender data not only compromises scientific integrity but also emboldens policies that marginalize vulnerable populations.^14,19^ These systemic inadequacies in administrative recognition can perpetuate discrimination and hinder equitable access to professional opportunities for gender-diverse individuals. In contrast, robust and inclusive data systems can serve as policy counterweights—affirming the legitimacy, presence, and contributions of gender-diverse providers.

## Conclusion

The ability for providers to authentically represent their identities is more than a bureaucratic detail. It is a matter of health equity, professional respect, and systemic accountability. The NPPES and NPI system, by virtue of their centrality in US health care infrastructure, must be reformed to reflect these values. By clearly distinguishing sex and gender, expanding identity options, providing educational support, and committing to regular updates, CMS can create a data system that is not only inclusive but exemplary. In doing so, it will send a powerful message: that equity in health care begins with equity in data.

## Abbreviations Used

CMS: Centers for Medicare and Medicaid Services
DEI: Diversity, equity, and inclusion
EHR: Electronic health record
HIPAA: Health Insurance Portability and Accountability Act
NPI: National Provider Identifier
NPPES: National Plan and Provider Enumeration System
US: United States
